# Yttrium Oxide Nanoparticles Moderate the Abnormal Cognitive Behaviors in Male Mice Induced by Silver Nanoparticles

**DOI:** 10.1155/2022/9059371

**Published:** 2022-04-26

**Authors:** Gasem Mohammad Abu-Taweel, Mohsen Ghaleb Al-Mutary, Hani Manssor Albetran

**Affiliations:** ^1^Department of Biology, College of Sciences, Jazan University, P.O. Box 2079, Jazan 45142, Saudi Arabia; ^2^Department of Biology, College of Science, Imam Abdulrahman Bin Faisal University, P. O. Box 383, Dammam 31113, Saudi Arabia; ^3^Basic and Applied Scientific Research Center (BASRC), Imam Abdulrahman Bin Faisal University, P.O. Box 1982, Dammam 31441, Saudi Arabia; ^4^Department of Physics, College of Science, Imam Abdulrahman Bin Faisal University, P.O. Box 1982, Dammam 31441, Saudi Arabia

## Abstract

Silver nanoparticles (Ag-NPs) have been used in medical, agricultural, and industrial purposes. Furthermore, NPs can cross the blood-brain barrier and encourage some effects on spatial learning and memory in organism. Here, we investigate the possible neurotoxicity of Ag-NPs with special emphasis on the neuroprotective impacts of yttrium-oxide nanoparticles (YO-NPs) in male mice. Male mice (*n* = 24) were weekly intraperitoneally injected for 35 days as the following; groups I, II, III, and IV received tap water (control), Ag-NPs (40 mg/kg), YO-NPs (40 mg/kg), and Ag-NPs/YO-NPs (40 mg/kg each), respectively. After that, animals were tested in shuttle box, Morris water-maze, and T-maze devices to evaluate the spatial learning and memory competence. Neurotransmitters and oxidative indices in the forebrain were estimated. According to behavioral studies, the male animals from the Ag-NP group presented worse memory than those in the control group. The biochemical changes after Ag-NP exposure were observed through increasing TBARS levels and decline in oxidative biomarkers (SOD, CAT, GST, and GSH) and neurotransmitters (DOP, SER, and AChE) in the forebrain of male mice compared to untreated animals. Interestingly, the animals treated with mixed doses of Ag-NPs and YO-NPs displayed improvements in behavioral tests, oxidative parameters, and neurotransmitters compared to males treated with Ag-NPs alone. In conclusion, the abnormal behavior related to learning and memory in male mice induced by Ag-NPs was significantly alleviated by YO-NPs. Specifically, the coinjection of YO-NPs with Ag-NPs moderates the disruption in neurotransmitters, oxidative indices of mice brains, which reflects on their cognitive behaviors.

## 1. Introduction

The global industrial revolution has introduced many innovative materials and devices that mainly depend on nanoparticles (NPs) [1]. Some NPs are used as food additives which could induce toxicity in the liver, digestive system, and gut microbiota changes [2]. Silver nanoparticles (Ag-NPs) are hugely used in water purification purposes, medical applications, food packages, antibacterial agents, cosmetics, and sunscreens [3]. The expansion of Ag-NPs elevates exposure in workers, consumers, and the environmental species. Ag-NPs are known to induce oxidative stress, DNA damage, cellular apoptosis, genotoxicity, and anatomical structures alternations in many organs [4–6]. The chronic treatment by Ag-NPs induced memory impairment and disrupted hippocampal synaptic plasticity in rats [7, 8]. In addition, the exposure by Ag-NPs for three days led to its accumulation in the duodenum and then transported to the liver, kidneys, and spleen through the circulatory system [9].

The blood-brain barrier (BBB) is a complex protective shield which considered the first obstacle to reaching the chemicals and drugs into the brain [10, 11]. However, the properties of Ag-NPs help them to penetrate the BBB, aggregate in brain tissues, and thereby induce neurotoxicity and affect neurological functions [12–17]. Exposing maternal rodents to Ag-NPs impaired spatial cognition of offspring [18, 19].

However, some NPs are an attractive therapeutic alternative to counteract the oxidative stress (OS) produced by some toxic materials [20, 21]. Metal oxide-based nanoparticles modulate the redox environment balance of tissues and enhance cell proliferation [22]. The in vivo and in vitro experiments have proven the anti-inflammatory and antioxidant properties of yttrium-oxide nanoparticles (YO-NPs) by attenuating fulminant hepatic failure and cell apoptosis [23]. The combination of YO-NPs and cerium oxide provided protection against brain apoptosis and lipid peroxidation when rats were exposed to lead [24]. Various studies have been focused on the potential neurobehavior and biochemistry toxicity of Ag-NPs in mice; however, no direct evidence investigated the protective effects of YO-NPs against Ag-NP toxicity in male mice. Therefore, this novelty study was to explore the potential role of YO-NPs in mitigating the neurobehavioral toxicity in male mice induced by Ag-NPs through learning and memory assessments and brain biochemical analysis.

## 2. Materials and Methods

### 2.1. Materials and Instrumentation of NP Examination

Scanning electron microscope (SEM, IRMC-INSPECT S50), transmission electron microscope (TEM, FEI Morgagni 268), and Rigaku Benchtop Miniflex X-ray diffractometer (XRD, with Cu–K*α* radiation) were used to characterize the Ag-NPs and YO-NPs (*M*_*w*_ = 225.81 and 107.87 g/mol, respectively; both Sigma-Aldrich, Inc., NSW, Australia).

### 2.2. Experimental Design and NP Treatments

**Figure 1:**
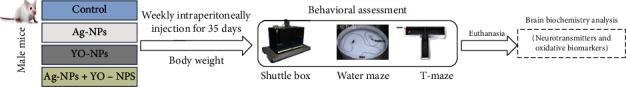
Experimental design of the effects of YO-NPs on neurobehavioral features, neurotransmitters, and oxidative indicators in the brain of male mice exposed to Ag-NPs. The animals were injected weekly for 35 days with PNs. After the exposure, neurobehavioral were assessed by shuttle box, Water Maze, and T-Maze tests. The animals were euthanized, and the hippocampus was collected for oxidative and neurotransmitter biochemistry analysis.

Before the experiment, 24 male Swiss–Webster strain mice (~75-days old, 25–35 g) were housed in an experimental room for one week to adapt to the 12-h dark–light cycle, and stable temperature (~25°C) with free access to food and water. Four groups of male mice with six animals per group were injected intraperitoneally every seven days. Groups I, II, III, and IV received tap water (control), Ag-NP (40 mg/kg), YO-NP (40 mg/kg), and Ag-YO-NP mixtures (40 mg/kg each), respectively. The brain biochemistry and neurobehavior were evaluated after 35 days of weekly injection (Figure 1). The concentrations of Ag-NPs and YO-NPs were selected based on the previously published studies described by Alarifi et al. [25] and Song et al. [23], respectively. This in vivo experimental study was approved by Jazan University, Jazan, Saudi Arabia, with the ethical number “REC-43/02/019.”

### 2.3. Body Weight

The animals were weighed every seven days, whereas the organs were weighed after sacrifice the animals.

### 2.4. Behavioral Evaluation

Spatial learning and memory competence were evaluated by shuttle box (Ugo Basile, Comerio, Varese, Italy), Morris water-maze (MWM), and T-maze (TM) devices according to the previous procedures [26].

#### 2.4.1. Shuttle Box Device (SB)

The active avoidance reactions were estimated in each male mouse via an automatic shuttle box connected with a printer. The number of crossings (NC), intertrial crossings (NIC), stimulated crossings (NSC), and reinforced crossings (NRC) were recorded. In addition, the latency to avoid shock (LAS) and total time to avoid shock (TAS) were counted.

#### 2.4.2. Morris Water-Maze Device (MWM)

MWM test was performed by a round pool filled with water (22 ± 1°C) and 500 ml milk in a 30 cm deep and had a little platform (1 cm under the water surface). Signs in four directions are placed around the MWM device. The mice should find the platform during the swimming with the help of the four signs. The mice are trained for three days. On the fourth day, the platform was removed for a probe test. The spent time (in sec) that animal remains in four places (probe test) and the time required (in sec) to find the platform was registered. If the mice could not find the platform within two minutes, they were placed on it for 30 sec forcibly.

#### 2.4.3. T-Maze Device (TM)

TM device was built of the major arm (100 × 10) and two lateral arms (40 × 10) in a T shape and a height of 20 cm. The mice food-deprived were tested to reach to the food placed in one of the lateral arms. The time (in sec) that the mice spent for reaching to the food and staying on it was recorded. The frequency and the spent time mice entered the food arm were counted.

### 2.5. Biochemical Studies

The animals were slaughtered after the completion of the behavioral experiments, and then, the animals' forebrains were extracted and frozen at -80°C until the examination time of the biochemical parameters. The concentrations of neurotransmitters and oxidative biomarkers in the forebrain were estimated according to our previous study [27]. Briefly, the forebrain tissues were homogenized in a solution consisting of 0.1 M of Perchloric acid +0.05% EDTA and centrifuged at 17,000 rpm for 5 min at 4°C. Then, the supernatants were filtered by 0.45 *μ*m pore filters. The neurotransmitters and oxidative parameters were analyzed by HPLC.

## 3. Statistical Analysis

By GraphPad Prism statistical program, the ANOVA method was used followed by Tukey's multiple comparisons test with significant value *P* ≤ 0.05[28].

## 4. Results

### 4.1. Morphological Study and NP XRD Analysis

**Figure 2:**
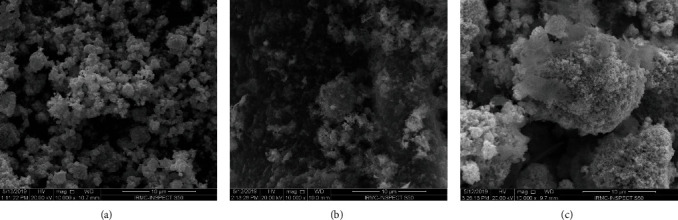
SEM micrographs of (a) Ag-NPs, (b) YO-NPs, and (c) nanoparticles mixture.

The NP morphology, size, and microstructure were studied by SEM and TEM. Typical SEM micrographs of the Ag-NP, YO-NP, and Ag-YO-NP mixtures in Figures 2(a)–2(c) show that the Ag-NPs were comprised of primary-particle agglomerates, whereas the YO-NP morphology was nebulous.

**Figure 3:**
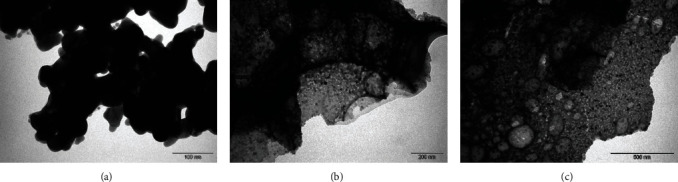
TEM micrographs of (a) Ag-NPs, (b) YO-NPs, and (c) nanoparticle mixture.

TEM confirmed the irregular agglomerate NP formation of the Ag-NPs, YO-NPs, and mixture (Figures 3(a)–3(c)) with a varying size as measured by using ImageJ® software (Version 1.48e, National Institutes of Health, USA). The YO-NP, Ag-NP, and the mixture mean sizes and standard deviations were 18 ± 5 nm, 19.5 ± 5 nm, and 14 ± 5 nm, respectively. The TEM image-contrast variations of the NP mixture in Figure 3(c) show an atomic-number variation; X-rays are dispersed more by the silver with a higher atomic number than the YO-NPs.

**Figure 4:**
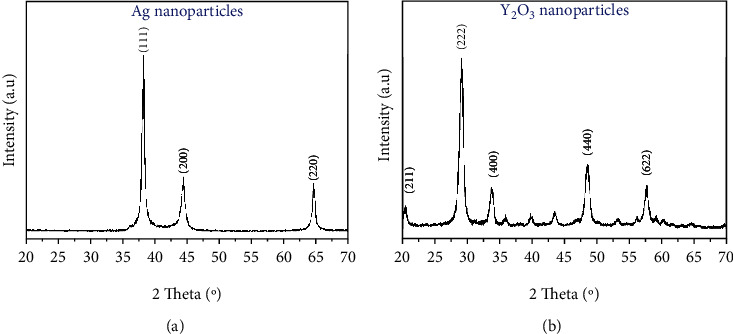
XRD patterns of (a) Ag-NPs and (b) YO-NPs.

XRD patterns of the Ag-NPs and YO-NPs (Figures 4(a) and 4(b), respectively) were obtained from the International Centre for Diffraction Data (ICDD) powder diffraction file database (Card No. 89-5591) and the Joint Committee on Powder Diffraction Standards (Card No. 04-0783). No second-phase peaks and impurities existed in the crystalline NPs. The average as determined by using Scherrer's equation [27–29] was used to calculate the average NP crystallite sizes (*L*) of the YO-NPs and Ag-NPs as 11.14 and 19.97 nm, respectively. Average particle diameters from TEM images provided comparable crystallite sizes.

### 4.2. Body and Organs Weights

**Figure 5:**
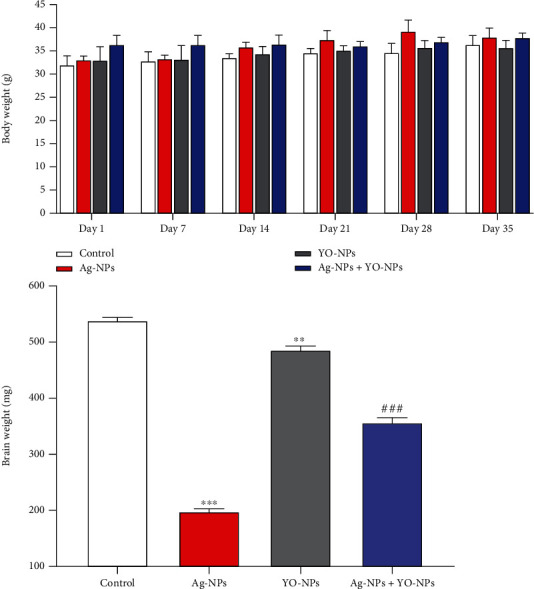
The body and brain weights of mice treated with Ag-NPs and YO-NPs alone or combination for 35 days.

There were no significant differences in body weight through the experiment between groups of male mice. Treated mice in the Ag-NP group had the lowest brain weights compared with those in the control group (*P* ≤ 0.001), whereas the brain weights of mice significantly increased in Ag-NP and YO-NP group compared to Ag-NP group (*P* ≤ 0.001) (Figure 5).

### 4.3. Learning and Memory Experiments

**Figure 6:**
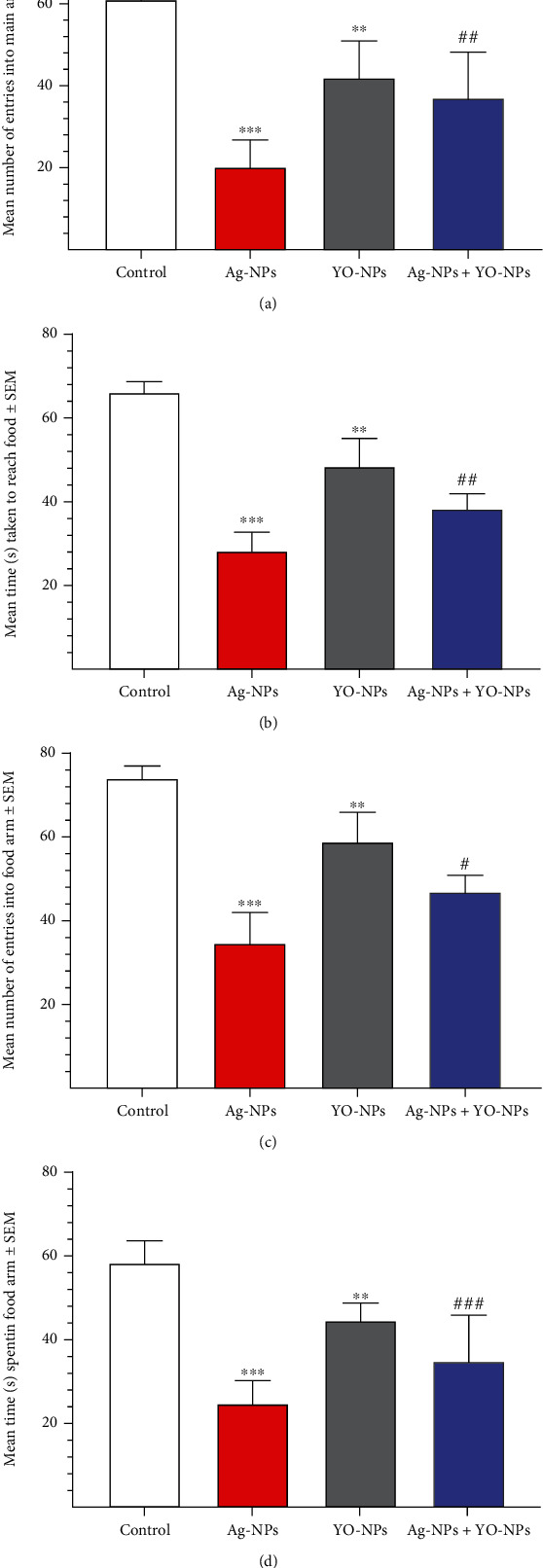
(a–d) The results of T-Maze experiment weekly treated mice with 40 mg/kg of Ag-NPs and YO-NPs alone or combination for 35 days. The symbols ^∗∗^ and ^∗∗∗^ mean a significant difference compared to the control group at *P* ≤ 0.01 and *P* ≤ 0.001, respectively. While the symbols ^#^, ^##^, and ^###^ mean a significant difference compared to Ag-NPs group at *P* ≤ 0.05, *P* ≤ 0.01, and *P* ≤ 0.001, respectively.

In T-maze experiment, the mice from the Ag-NP group presented worse memory than those males in the control group (*P* ≤ 0.001). This was significantly observed (*P* ≤ 0.001) in treated males through the mean number of entries into the main arm and food arm and also in the results of the time taken to reach and to spend in the food arm (Figure 6). However, the treated mice by YO-NP alone or with Ag-NPs have shown significant (at least at *P* ≤ 0.05 level) improvement in their learning and memory compared to Ag-NP group.

**Figure 7:**
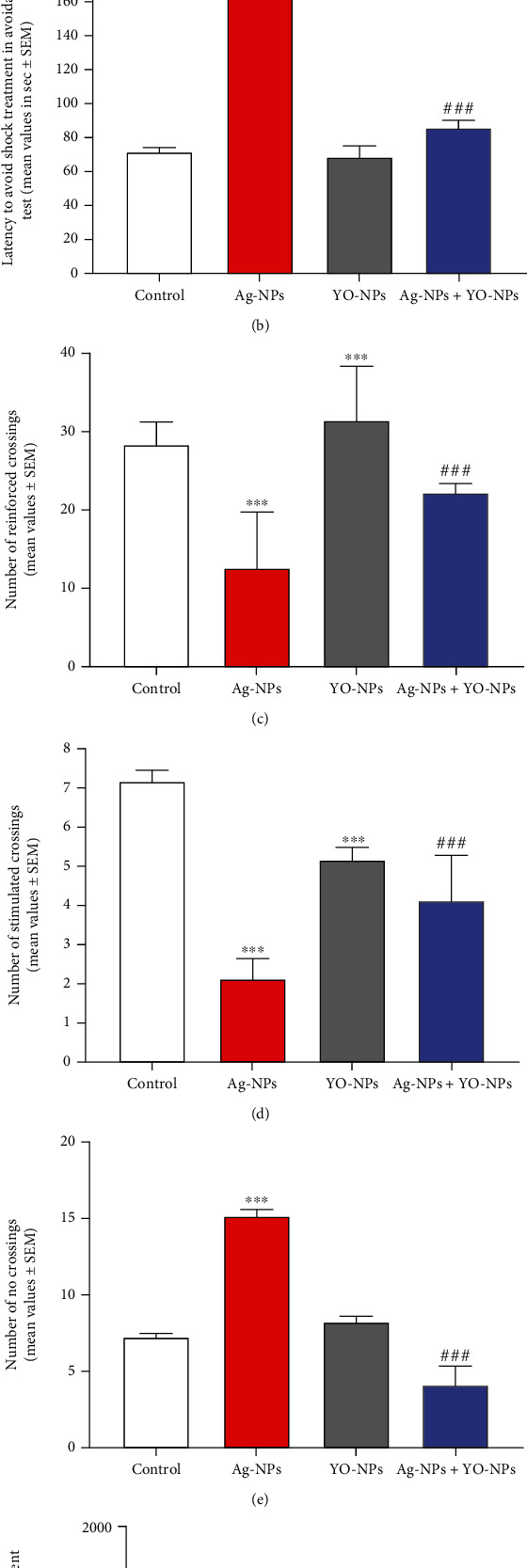
(a–f) The active avoidance reactions in male mice treated weekly with 40 mg/kg of Ag-NPs and YO-NPs alone or combination for 35 days. The symbols ^∗∗∗^ and ^###^ mean a significant difference compared to the control and Ag-NPs groups, respectively, at *P* ≤ 0.001.

In shuttle box test, the mice exposed to Ag-NPs showed a significant decrease (*P* ≤ 0.001) in the number of intertrial, stimulated, and reinforced crossings after treatment as compared to the control group. The males exposed to Ag-NPs were poor learners and took more time responding to the shock treatment and less number of crossings between two chambers as compared to untreated males. There were no negative effects of YO-NPs throughout shuttle box testing parameters in animals; however, there were ameliorated effects by YO-NP against Ag-NP toxicity (Figure 7).

**Figure 8:**
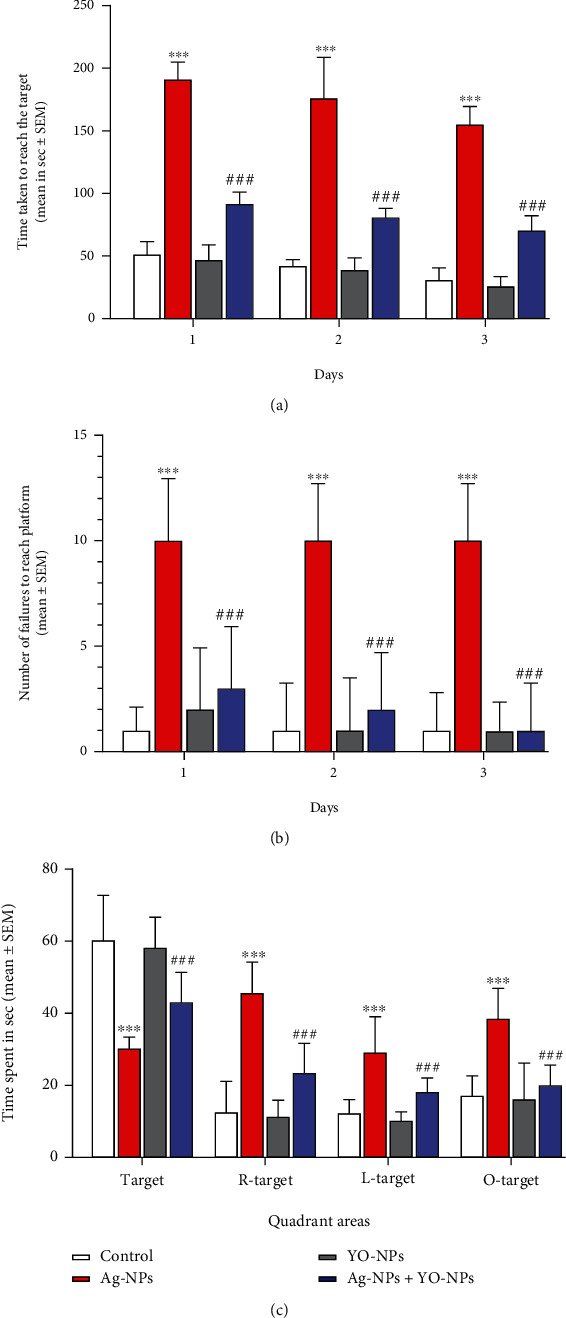
(a–c) The results of water-maze experiment on male mice treated weekly with 40 mg/kg of Ag-NPs and YO-NPs alone or combination for 35 days. The ^∗∗∗^ and ^###^ symbols mean a significant difference compared to the control and Ag-NPs groups, respectively, at *P* ≤ 0.001.

In MWM task (Figure 8), mice with Ag-NP treatment exhibited longer escape latencies to reach the target as compared with the control group (*P* ≤ 0.001). The number of failing trials to arrive at the target was higher (*P* ≤ 0.001) in Ag-NP-treated mice as compared to the control group on all trial days. The probe test experiments displayed that Ag-NPs exposed males took more time in the R-target, L-target, and O-target quadrants than the platform quadrant as compared to the control group (*P* ≤ 0.001) to find the platform. However, the Ag-NP and YO-NP groups displayed amelioration (*P* ≤ 0.001) in behavior over the training days.

### 4.4. Biochemical Parameters in the Brain of Male Mice

**Figure 9:**
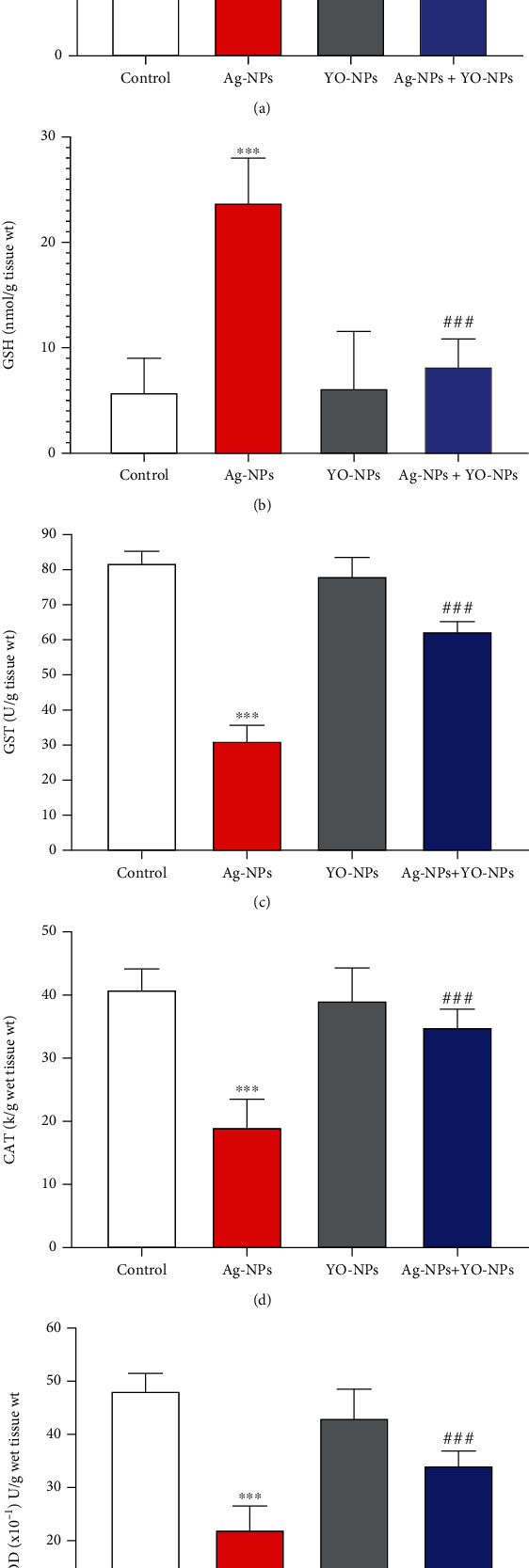
(a–e) The oxidative markers concentrations in the brain of male mice treated weekly with 40 mg/kg of Ag-NPs and YO-NPs alone or combination for 35 days. The symbols ^∗∗∗^ and ^###^ mean a significant difference compared to the control and Ag-NPs groups, respectively, at (*P* ≤ 0.001).

**Figure 10:**
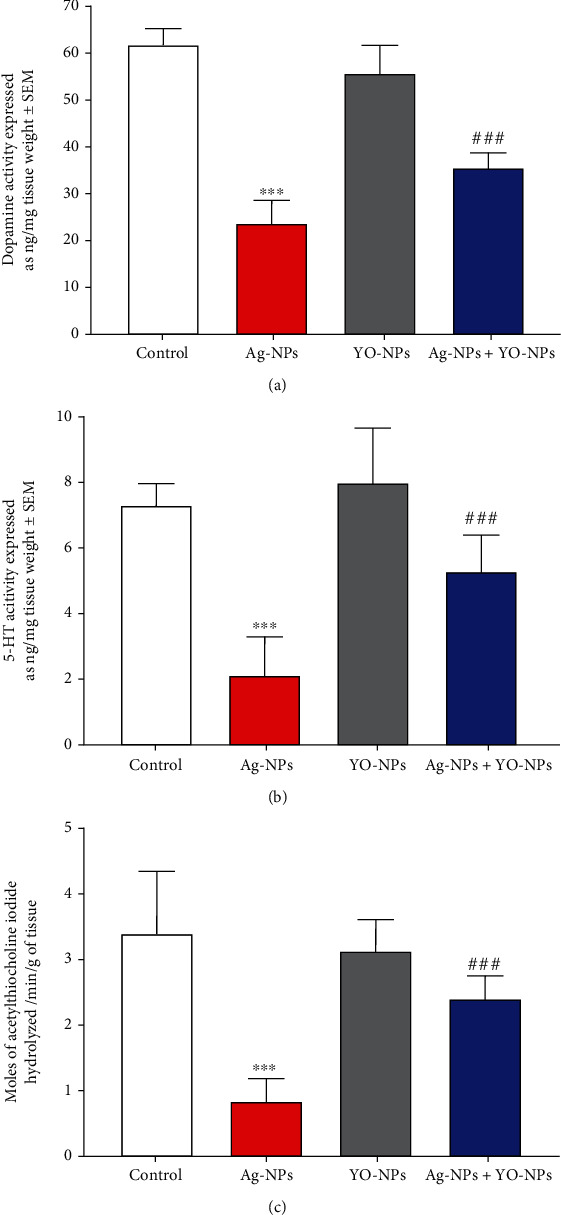
(a–c) The neurotransmitters levels in the brain of male mice treated weekly with 40 mg/kg of Ag-NPs and YO-NPs alone or combination for 35 days. The symbols ^∗∗∗^ and ^###^ mean a significant difference compared to the control and Ag-NP groups, respectively, at (*P* ≤ 0.001).

The major effect of Ag-NPs was significant (*P* ≤ 0.001) by increasing the levels of TBARS in the hippocampus of mice compared to untreated animals (Figure 9). Moreover, Ag-NPs augmented oxidative stress in the hippocampus of animals as observed by significant (*P* ≤ 0.001) decrease levels of oxidative parameters (SOD, CAT, GST, and GSH) and neurotransmitters (DOP, SER, and AChE) levels compared to the control group (Figure 10). However, YO-NPs improved (*P* ≤ 0.001) these parameters in the brain of male mice exposed to Ag-NPs.

## 5. Discussion

Daily exposure to Ag-NPs has become intimate through routine uses such as household appliances, food industries, cosmetics, and drug delivery [30]. Many studies have demonstrated the toxicity of Ag-NPs on an organism's brain [8, 31, 32]. Therefore, many materials had to be invented to mitigate the side effects of Ag-NP toxicity. In this study, the effects of Ag-NPs alone or combined with YO-NPs on the cognitive abilities of male mice under weekly intraperitoneally exposure for 35 days were investigated using shuttle box, MWM, and TTM devices. Moreover, the brain function was also examined through neurotransmitters levels and oxidative status in the forebrain. We reported on neurobehavioral dysfunctions and biochemical disorders in the brain after Ag-NPs exposure. Furthermore, we found that YO-NPs offered a therapeutic role to emaciate the adverse effects of Ag-NPs on brain function.

Ag-NPs can pass the blood-brain barrier via passive diffusion or endocytosis [31, 33–35] and also may be crossed by transsynaptic processes [35]. Thus, they disrupt the antioxidant defenses, cause inflammation, and induce apoptosis in the brain [14]. Numerous studies support the proof that exposure to Ag-NP consequence impairment in cognition behaviors in various organisms [31, 36–39]. According to this experiment, the weekly injection of Ag-NPs for 35 days produced learning and memory deficits in male mice as we observed in SB, MWM, and TM performance. Liu et al. [8] reported that the overproduction of ROS may initiate an oxidative stress which may be the reason of neurotoxicity made by Ag-NPs. Our results showed that the males treated with Ag-NPs produced high amounts of TBARS and low levels of SOD, CAT, GST, and GSH compared to untreated males. Oxidative stress can disrupt the antioxidant defenses in the brain, leading to a decline in neurotransmitters, and cause some memory deficits [40].

Neurotransmitters are liable for signal transfer from neurons to target cells and for the regulation of a diversity of behavioral processes [41, 42]. DOP controls numerous behavioral processes, such as locomotion, emotional responses, memory traces, and social interactions [43, 44]. Furthermore, Hritcu et al. [45] and Yuan et al. [46] reported the relationship between hippocampal serotonin and acetylcholine levels and memory efficiency in rats. Herein, the abnormal behaviors related to memory and learning synchronized with the decline in neurotransmitters (DOP, SER, and AChE) levels in the forebrain. Previous studies mentioned the connection of neurotransmitters disruption and their effects on spatial cognition weakness in Ag-NP-treated rats [8, 47]. The Ag-NP exposure resulted in some effects on synaptic proteins, cytoskeleton, and mitochondria integrity [48].

YO-NPs have been demonstrated to be a powerful element that can be applied for many biomedicine purposes [49]. We noted in our previous experiment that YO-NPs moderated the toxic effects of Ag-NPs on testicular function by improving oxidative parameters and decreasing apoptotic cells [50]. Schubert et al. [51] mentioned the ability of the YO-NPs to protect nerve cells from oxidative stress. In addition, Navaei-Nigjeh et al. [52] confirmed that YO-NPs raised the total antioxidant capacity and minimized lipid peroxidation and reactive oxygen species (ROS) in brain rats exposed to diazinon. The ROS production during oxidative stress can damage mitochondria function, impair antioxidant defense, disrupt cellular metabolism, modify neurotransmitters releasing which cause synaptic failure in the brain, and finally, drive some cognition disorders [53]. In this study, the coinjection of YO-NPs with Ag-NPs improved oxidative parameters and neurotransmitters in the brain. This healthy state was reflected through the neurobehavioral tests of mice by using MWM, T-M, and SB devices. We suggest that YO-NPs can penetrate the nerve cells, work as antioxidants by scavenging free radicals and enhance endogenous antioxidant activities which may maintain ATP production and protect cellular membranes and organelles.

## 6. Conclusions

Exposure to Ag-NPs promotes some abnormal alterations in brain neurotransmitters (DOP, SER, and AChE), oxidative indices (TBARS, SOD, CAT, GST, and GSH), and cognitive behaviors (according to MWM, T-M, and SB tasks) in male mice. Interestingly, the coinjection of YO-NPs with Ag-NPs mitigates these effects.

## Data Availability

All datasets generated for this study are included in the article.

## Abbreviations

Ag-NPs: Silver nanoparticles
YO-NPs: Yttrium-oxide nanoparticles
BBB: Blood-brain barrier
OS: Oxidative stress
SEM: Scanning electron microscope
TEM: Transmission electron microscope
XRD: X-ray diffractometer
IRB: Institutional Review Board
MWM: Morris water-maze
TM: T-maze
SB: Shuttle box device
NC: Number of crossings
NIC: Number of intertrial crossings
NSC: Number of stimulated crossings
NRC: Number of reinforced crossings
LAS: Latency to avoid shock
TAS: Total time to avoid shock
ICDD: International Centre for Diffraction Data
*L*: Crystallite sizes
SOD: Superoxide dismutase
CAT: Catalase
GST: Glutathione S-transferase
GSH: Glutathione
DOP: Dopamine
SER: Serotonin
AChE: Acetylcholinesterase.

## References

[1] Jeevanandam J., Barhoum A., Chan Y. S., Dufresne A., Danquah M. K. (2018). Review on nanoparticles and nanostructured materials: history, sources, toxicity and regulations. *Beilstein Journal of Nanotechnol*.

[2] Medina-Reyes E. I., Rodríguez-Ibarra C., Déciga-Alcaraz A., Díaz-Urbina D., Chirino Y. I., Pedraza-Chaverri J. (2020). Food additives containing nanoparticles induce gastrotoxicity, hepatotoxicity and alterations in animal behavior: the unknown role of oxidative stress. *Food and Chemical Toxicology*.

[3] Chernousova S., Epple M. (2013). Silver as antibacterial agent: ion, nanoparticle, and metal. *Angewandte Chemie International Edition*.

[4] Ahamed M., Alsalhi M. S., Siddiqui M. K. (2010). Silver nanoparticle applications and human health. *Clinica Chimica Acta*.

[5] Al-Doaiss A. A., Jarrar Q., Alshehri M., Jarrar B. (2020). In vivostudy of silver nanomaterials’ toxicity with respect to size. *Toxicology and Industrial Health*.

[6] Rodriguez-Garraus A., Azqueta A., Vettorazzi A., López de Cerain A. (2020). Genotoxicity of silver nanoparticles. *Nanomaterials*.

[7] Wesierska M., Dziendzikowska K., Gromadzka-Ostrowska J. (2018). Silver ions are responsible for memory impairment induced by oral administration of silver nanoparticles. *Toxicology Letters*.

[8] Liu Y., Guan W., Ren G., Yang Z. (2012). The possible mechanism of silver nanoparticle impact on hippocampal synaptic plasticity and spatial cognition in rats. *Toxicology Letters*.

[9] Narciso L., Coppola L., Lori G. (2020). Genotoxicity, biodistribution and toxic effects of silver nanoparticles after _in vivo_ acute oral administration. *NanoImpact*.

[10] Pardridge W. M. (2007). Blood-brain barrier delivery. *Drug Discovery Today*.

[11] Reichel A. (2009). Addressing central nervous system (CNS) penetration in drug discovery: basics and implications of the evolving new concept. *Chemistry & Biodiversity*.

[12] Al Gurabi M. A., Ali D., Alkahtani S., Alarifi S. (2015). In vivo DNA damaging and apoptotic potential of silver nanoparticles in Swiss albino mice. *Therapy*.

[13] Lee J. H., Kim Y. S., Song K. S. (2013). Biopersistence of silver nanoparticles in tissues from Sprague-Dawley rats. *Particle and Fibre Toxicology*.

[14] Lebda M. A., Sadek K. M., Tohamy H. G. (2018). Potential role of *α*-lipoic acid and _Ginkgo biloba_ against silver nanoparticles-induced neuronal apoptosis and blood-brain barrier impairments in rats. *Life Sciences*.

[15] Safari M., Arbabi Bidgoli S., Rezayat S. M. (2016). Differential neurotoxic effects of silver nanoparticles: a review with special emphasis on potential biomarkers. *Nanomedicine Journal*.

[16] Ahmed M. M., Hussein M. M. A. (2017). Neurotoxic effects of silver nanoparticles and the protective role of rutin. *Biomedicine & Pharmacotherapy*.

[17] Engin A. B., Engin A. (2019). Nanoparticles and neurotoxicity: dual response of glutamatergic receptors. *Progress in Brain Research*.

[18] Wu J., Yu C., Tan Y. (2015). Effects of prenatal exposure to silver nanoparticles on spatial cognition and hippocampal neurodevelopment in rats. *Environmental Research*.

[19] Ghaderi S., Tabatabaei S. R., Varzi H. N., Rashno M. (2015). Induced adverse effects of prenatal exposure to silver nanoparticles on neurobehavioral development of offspring of mice. *The Journal of Toxicological Sciences*.

[20] Karakoti A., Singh S., Dowding J. M., Seal S., Self W. T. (2010). Redox-active radical scavenging nanomaterials. *Chemical Society Reviews*.

[21] Ferreira C. A., Ni D., Rosenkrans Z. T., Cai W. (2018). Scavenging of reactive oxygen and nitrogen species with nanomaterials. *Nano Research*.

[22] Augustine R., Mathew A. P., Sosnik A. (2017). Metal oxide nanoparticles as versatile therapeutic agents modulating cell signaling pathways: linking nanotechnology with molecular medicine. *Applied Materials Today*.

[23] Song X., Shang P., Sun Z. (2019). Therapeutic effect of yttrium oxide nanoparticles for the treatment of fulminant hepatic failure. *Nanomedicine*.

[24] Hosseini A., Sharifi A. M., Abdollahi M. (2015). Cerium and yttrium oxide nanoparticles against lead-induced oxidative stress and apoptosis in rat hippocampus. *Biological Trace Element Research*.

[25] Alarifi A., Ali D., Al Ghurabi M. A., Alkahtani S. (2017). Determination of nephrotoxicity and genotoxic potential of silver nanoparticles in Swiss albino mice. *Toxicological & Environmental Chemistry*.

[26] Abu-Taweel G. M., Al-Mutary M. G. (2020). Pomegranate juice rescues developmental, neurobehavioral and biochemical disorders in aluminum chloride-treated male mice. *Journal of Trace Elements in Medicine and Biology*.

[27] Low I. M., Albetran H., Prida V. M., Vega V., Manurung P., Ionescu M. (2013). A comparative study on crystallization behavior, phase stability, and binding energy in pure and Cr-doped TiO2 nanotubes. *Journal of Materials Research*.

[28] Albetran H. M. (2020). Thermal expansion coefficient determination of pure, doped, and co-doped anatase nanoparticles heated in sealed quartz capillaries using _in-situ_ high-temperature synchrotron radiation diffraction. *Heliyon*.

[29] Albetran H. (2021). Investigation of the morphological, structural, and vibrational behaviour of graphite nanoplatelets. *Journal of Nanomaterials*.

[30] Jaswal T., Gupta J. (2021). A review on the toxicity of silver nanoparticles on human health. *Materials Today: Proceedings*.

[31] Greish K., Alqahtani A. A., Alotaibi A. F. (2019). The effect of silver nanoparticles on learning, memory and social interaction in BALB/C mice. *International Journal of Environmental Research and Public Health*.

[32] Dąbrowska-Bouta B., Zięba M., Orzelska-Górka J. (2016). Influence of a low dose of silver nanoparticles on cerebral myelin and behavior of adult rats. *Toxicology*.

[33] Hoet P. H., Brüske-Hohlfeld I., Salata O. V. (2004). Nanoparticles - known and unknown health risks. *Journal of Nanobiotechnology*.

[34] Trickler W. J., Lantz S. M., Murdock R. C. (2010). Silver nanoparticle induced blood-brain barrier inflammation and increased permeability in primary rat brain microvessel endothelial cells. *Toxicological Sciences*.

[35] Gonzalez-Carter D. A., Leo B. F., Ruenraroengsak P. (2017). Silver nanoparticles reduce brain inflammation and related neurotoxicity through induction of H_2_S-synthesizing enzymes. *Scientific Reports*.

[36] Oberdörster G., Sharp Z., Atudorei V. (2004). Translocation of inhaled ultrafine particles to the brain. *Inhalation Toxicology*.

[37] Fu C. W., Horng J. L., Tong S. K. (2021). Exposure to silver impairs learning and social behaviors in adult zebrafish. *Journal of Hazardous Materials*.

[38] Young A., Protheroe A., Lukowiak K. (2017). Silver nanoparticles alter learning and memory formation in an aquatic organism, _Lymnaea stagnalis_. *Environmental Pollution*.

[39] Antsiferova A., Kopaeva M., Kashkarov P. (2018). Effects of prolonged silver nanoparticle exposure on the contextual cognition and behavior of mammals. *Materials*.

[40] Haddadi M., Jahromi S. R., Sagar B. K., Patil R. K., Shivanandappa T., Ramesha S. R. (2014). Brain aging, memory impairment and oxidative stress: a study in Drosophila melanogaster. *Behavioural Brain Research*.

[41] García-Cazorla A., Artuch R., Rosenberg R. N., Pascual J. M. (2015). Chapter 63- Neurotransmitter disorders. *Rosenberg's Molecular and Genetic Basis of Neurological and Psychiatric Disease (Fifth Edition)*.

[42] Wideman C. E., Jardine K. H., Winters B. D. (2018). Involvement of classical neurotransmitter systems in memory reconsolidation: focus on destabilization. *Neurobiology of Learning and Memory*.

[43] Wise R. A. (2004). Dopamine, learning and motivation. *Nature Reviews Neuroscience*.

[44] Beaulieu J. M., Gainetdinov R. R. (2011). The physiology, signaling, and pharmacology of dopamine receptors. *Pharmacological Reviews*.

[45] Hritcu L., Clicinschi M., Nabeshima T. (2007). Brain serotonin depletion impairs short-term memory, but not long-term memory in rats. *Physiology & Behavior*.

[46] Yuan Y., Shan X., Men W. (2020). The effect of crocin on memory, hippocampal acetylcholine level, and apoptosis in a rat model of cerebral ischemia. *Biomedicine & Pharmacotherapy*.

[47] Hadrup N., Loeschner K., Mortensen A. (2012). The similar neurotoxic effects of nanoparticulate and ionic silver in vivo and in vitro. *Neurotoxicology*.

[48] Xu F., Piett C., Farkas S., Qazzaz M., Syed N. I. (2013). Silver nanoparticles (AgNPs) cause degeneration of cytoskeleton and disrupt synaptic machinery of cultured cortical neurons. *Molecular Brain*.

[49] Rajakumar G., Mao L., Bao T. (2021). Yttrium oxide nanoparticle synthesis: an overview of methods of preparation and biomedical applications. *Applied Sciences*.

[50] Abu-Taweel G. M., Albetran H. M., Al-Mutary M. G., Ahmad M., Low I. M. (2021). Alleviation of silver nanoparticle-induced sexual behavior and testicular parameters dysfunction in male mice by yttrium oxide nanoparticles. *Toxicology Reports*.

[51] Schubert D., Dargusch R., Raitano J., Chan S. W. (2006). Cerium and yttrium oxide nanoparticles are neuroprotective. *Biochemical and Biophysical Research Communications*.

[52] Navaei-Nigjeh M., Khaksar M. R., Rahimifard M., Baeeri M., Abdollahi M. (2017). Evaluation of oxidative stress biomarkers in rat brain exposed to diazinon and yttrium oxide nanoparticles. *Toxicology Letters*.

[53] Tonnies E., Trushina E. (2017). Oxidative stress, synaptic dysfunction, and Alzheimer's disease. *Journal of Alzheimer's disease: JAD*.

