# Correction to “Yttrium Oxide Nanoparticles Moderate the Abnormal Cognitive Behaviors in Male Mice Induced by Silver Nanoparticles”

**DOI:** 10.1155/omcl/9857537

**Published:** 2026-04-17

G. M. Abu‐Taweel, M. G. Al‐Mutary, and H. M. Albetran, “Yttrium Oxide Nanoparticles Moderate the Abnormal Cognitive Behaviors in Male Mice Induced by Silver Nanoparticles,” *Oxidative Medicine and Cellular Longevity*, 2022, 9059371, https://doi.org/10.1155/2022/9059371.

In the article, there is an error in Figure 2a, which is duplicated from a prior publication of the authors [1]. The correct Figure 2 is below:

**Figure 2:**
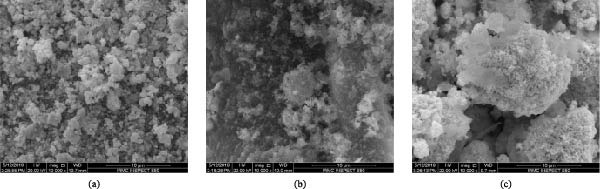
SEM micrographs of (a) Ag‐NPs, (b) YO‐NPs, and (c) nanoparticles mixture.

Additionally, there is an error in the molecular weights described in Section 2.1:

• “Scanning electron microscope (SEM, IRMC‐INSPECT S50), transmission electron microscope (TEM, FEI Morgagni 268), and Rigaku Benchtop Miniflex X‐ray diffractometer (XRD, with Cu–Kα radiation) were used to characterize the Ag‐NPs and YO‐NPs (M w = 225:81 and 107.87 g/mol, respectively; both Sigma‐Aldrich, Inc., NSW, Australia).”

Should read:

“Scanning electron microscope (SEM, IRMC‐INSPECT S50), transmission electron microscope (TEM, FEI Morgagni 268), and Rigaku Benchtop Miniflex X‐ray diffractometer (XRD, with Cu–Kα radiation) were used to characterize the Ag‐NPs and YO‐NPs (M w = 107.87 g/mol and 225.81 g/mol, respectively; both Sigma‐Aldrich, Inc., NSW, Australia).”

We apologize for these errors.
